# The classification of flash visual evoked potential based on deep learning

**DOI:** 10.1186/s12911-023-02107-5

**Published:** 2023-01-19

**Authors:** Na Liang, Chengliang Wang, Shiying Li, Xin Xie, Jun Lin, Wen Zhong

**Affiliations:** 1 College of Computer Science, Chongqing University, Chongqing, China; 2 Department of Ophthalmology, Xiang’an Hospital of Xiamen University, Xiamen University, Xiamen, China; 3 Department of Ophthalmology, Eye Institute of Xiamen University, Xiamen, China; 4 Department of Ophthalmology, Yongchuan People’s Hospital of Chongqing, Chongqing, China; 5 Chongqing Health Statistics Information Center, Chongqing, China

**Keywords:** Deep learning, FVEP, Out-of-distribution detection, Convolutional neural networks

## Abstract

**Background:**

Visual electrophysiology is an objective visual function examination widely used in clinical work and medical identification that can objectively evaluate visual function and locate lesions according to waveform changes. However, in visual electrophysiological examinations, the flash visual evoked potential (FVEP) varies greatly among individuals, resulting in different waveforms in different normal subjects. Moreover, most of the FVEP wave labelling is performed automatically by a machine, and manually corrected by professional clinical technicians. These labels may have biases due to the individual variations in subjects, incomplete clinical examination data, different professional skills, personal habits and other factors. Through the retrospective study of big data, an artificial intelligence algorithm is used to maintain high generalization abilities in complex situations and improve the accuracy of prescreening.

**Methods:**

A novel multi-input neural network based on convolution and confidence branching (MCAC-Net) for retinitis pigmentosa RP recognition and out-of-distribution detection is proposed. The MCAC-Net with global and local feature extraction is designed for the FVEP signal that has different local and global information, and a confidence branch is added for out-of-distribution sample detection. For the proposed manual features,a new input layer is added.

**Results:**

The model is verified by a clinically collected FVEP dataset, and an accuracy of 90.7% is achieved in the classification task and 93.3% in the out-of-distribution detection task.

**Conclusion:**

We built a deep learning-based FVEP classification algorithm that promises to be an excellent tool for screening RP diseases by using FVEP signals.

## Introduction

It is generally accepted that the eye is the most important sensory organ in the human body, and most external information comes from the eye. However, eye disease is caused by increased optic nerve injury, leading to poor vision and even blindness. Retinitis pigmentosa (RP) is one of the most severe optic nerve injury diseases, with a frequency of 1 in 3000–5000 people worldwide [1]. Current screening methods for RP include visual electrophysiology and genetic testing [2]. Visual electrophysiology is an objective visual function examination widely used in clinical work that can objectively evaluate the function of the retina, or optic nerve, and objectively reflect the corresponding changes of the disease according to the waveform change. Different diseases have their own characteristic visual electrophysiological examination results. In clinics, a diagnosis requires a combination of clinical manifestations, fundus fluorescence angiography, optical coherence tomography genetic testing and visual electrophysiology.

**Fig. 1:**
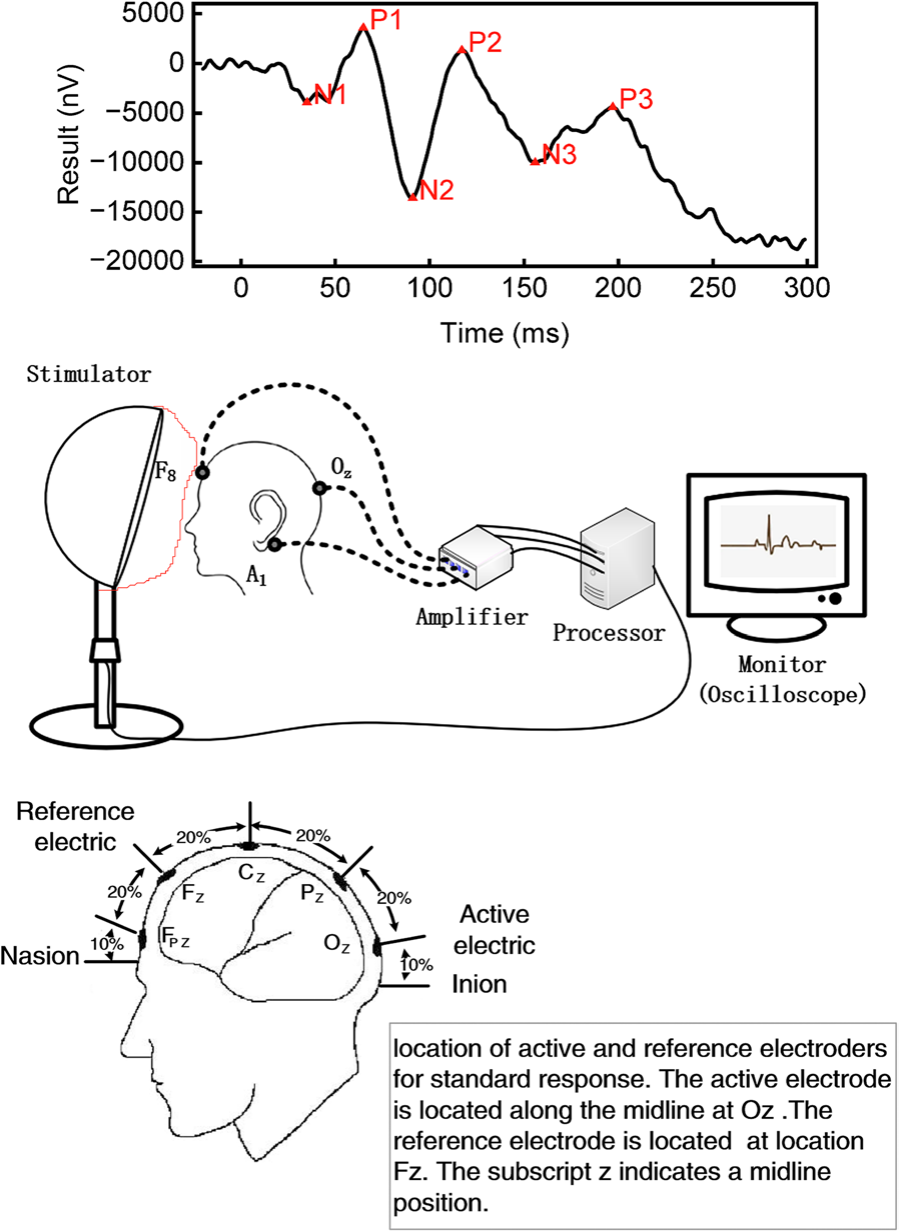
A comparison of the diagnostic process between doctors and AI

In clinical practice, doctors mainly use genetic tests to confirm the diagnosis of RP disease, however the tests are expensive and take a long time to perform, and are not suitable for primary screening. The visual evoked potential (VEP) is commonly adopted to detect RP, which is an important reference for the functional integrity of the visual system. Flash VEP (FVEP) is a sensitive test for optic nerve injury, and as a rapid and inexpensive test is an important tool for the primary screening of RP diseases. Moreover, FVEP is adopted not only in the field of ophthalmology but also is a good tool in the diagnosis and treatment of Alzheimer’s disease [3], multiple sclerosis and tumours in the pterygoid saddle area [4]. The diagnosis of RP and other diseases related to optic nerve injury depends on the labelling and identification of the FVEP signals by professional doctors. As shown in Fig. 1, doctors use domain knowledge to label 6 feature points in the FVEP signal for a diagnosis of the disease. Unfortunately, these labels and identifications are time-consuming and very dependent on doctor experience. In addition, the lack of specialists familiar with electrophysiology, and the lack of knowledge about RP disease, make it difficult to initially screen for RP disease by FVEP. Computer-assisted analysis can solve these problems.

### Electrode location

Visual evoked potential (VEP) is a bioelectric activity produced by visual stimulation of the retinal photoreceptors, such as from light, which is transmitted through the retina, optic nerve, optic radiation and finally to the occipital cortex. The instrument receives bioelectrical signals through skin contact electrodes on the head and face, enhances the signals through an amplifier, and computer analysis converts the electrical signals into a graphic form. We used the Espion E2 visual electrophysiological examiner from the USA to collect the patient data. The skin was fully cleaned before the examination, and the contact electrodes were coated with conductive paste. See Fig. 1. The anterior-posterior midline of the vertex was determined according to the nasal root and the occipital ridge. The detection electrode was placed on the occipital scalp at the OZ above the visual cortex, and the reference electrode was placed at the FZ [5]. A separate electrode was used as the grounding electrode, and common locations include the forehead, the vertex (CZ) mastoid, the earlobe or the line connecting the bilateral earlobes.

### Parameter setting

Electroretinography was performed with an Espion E3 visual electrophysiology testing system(Diagnosys LLC, Lowell, MA, USA). Flash VEPs were elicited using the Espion E3 Ganzfeld ColorDome stimulator. White flash stimuli (6500K) were used, which were delivered on no background. Flash strength of $3\,\hbox{cd s}/\hbox{m}^{2}$ was chosen. The stimulus duration was 4 ms. The sweep pre-trigger time was 20 ms and the sweep post-trigger time was 300 ms The flashes were delivered at 1 Hz, and the means of 70 responses were recorded and repeated at least twice. The signals were amplified with a band-pass from 0.312 to 100 Hz.

### Flash stimulation

The parameter for flash stimulation is a very brief (5 ms) flash evocation, and the flash frequency should be 1 flash per second (i.e., 1.0 Hz, in the range 0.9–1.1 Hz) [6]. DC amplifiers or AC coupled amplifiers with a minimum input impedance of 10 m in the 50–60 Hz range can be used. In compliance with current medical safety standards, the amplifier system must be insulated from the patient. It is difficult to obtain satisfactory recordings using these filter settings when severe electromagnetic interference from the stimulus monitor is encountered. Ideally, such interfering signals should be eliminated by shielding or adjusting the equipment.

### Sampling rate and signal acquisition

The sampling rate of Southwest Hospital is 1000 Hz. Sweep pre-trigger time 20 ms,sweep post-trigger time 300 ms, 80 sweeps per result. Filter band: band-pass from 0.312 to 100 Hz,and the number of superimpositions can be between 64 and 128 times, depending on the fixation. According to the characteristics of the FVEP signal, the appropriate IMF components (IMF3, IMF4, IMF5) are selected for reconstruction, and the corresponding IMF components are selected for reconstruction to obtain the FVEP signal after effective denoising, which can realize the single extraction of the FVEP signal.

### Processing steps

The research team took an analog electrical signal and converted it directly into a digital signal through a 32-bit analog-to-digital signal converter. The processing steps are as follows: 1. Use the detector to record the single FVEP signal of the left and right visual pathways, and select 1 data in the left and right visual pathways as the original data of the single FVEP signal; 2. Use the empirical modal decomposition method to obtain each MF component of the original FVEP signal; 3. Then select the MF component you study according to the frequency band range of the FVEP signal; 4. Use the selected each MF component to reconstruct the signal, so as to obtain the FVEP signal after noise removal, and realize the single extraction of FVEP.

### Reporting and Marking

The report generally contains the following recording parameters: the filter settings and the positions of the anode (recording), cathode (reference) and ground electrodes. The waveform traces should clearly indicate the polarity, the time reference scale in milliseconds, and the amplitude in microvolts. After obtaining this report, physicians need to apply their expertise in conjunction with the FVEP signal points to mark them as a way to diagnose RP disease. However, this obviously relies on the physician’s experience and system knowledge and is time-consuming. This process presents a challenge for physicians who lack experience and system knowledge, and computer-aided analysis can solve these problems.

This paper introduces an RP disease recognition model that combines domain knowledge with an out-of-distribution detection ability. First, this paper proposes a neural network model to extract the global features and local features to achieve higher accuracies. Then, this paper adopts temporal, statistical and spectral methods to extract features with domain knowledge to further improve the accuracy. Finally, this paper introduces a confidence branch to solve the out-of-distribution sample detection problem.

The main contributions of this paper are as follows:

- We propose a neural network that combines global convolutions with local convolutions.
- The FEVP signal features are extracted with domain knowledge.
- The confidence branches method is adopted for out-of-distribution detection.

## Related research

Currently, machine learning methods are widely used in medical fields, for example for medical image classification [7], and COVID-19 prediction [8]. Much research in recent years has focused on the auto recognition of FVEP signals by machine learning methods.

Qiao introduced FVEP into the detection of neurological functions during the central neurooncology procedure, collected FVEP signs at multiple points in time, and then converted the FVEP sequence into a two-dimensional image for classification [4]. They first proposed a simple three-layer convolutional neural network using CAM (Class Activation Mapping) for visualization and analysis and found that the activation regions were mainly between P2–N3–P3 [9]. In addition, they proposed a CNN-LSTM network, where images are first passed through the CNN network to extract the visual features and then output to the LSTM to extract the temporal features. Finally, in the test set, the sensitivities of the three categories were 92.6%, 78.9% and 83.7%, and the specificities were 80.5%, 93.3% and 100%, respectively. Waytowich proposed the Compact Convolutional Neural Network, which adopted deep separable convolution instead of normal convolution to reduce the dimensionality of the data and reduced the number of parameters while efficiently extracting the frequency-specific information [10]. In addition, it showed a significant performance improvement compared with the traditional canonical correlation analysis-based classification algorithms. Parthiban et al. [11] proposed a novel hierarchical attentional neural network, which included two subnetworks; one subnetwork focused on the minutiae features, and the other subnetwork acquired a global view and finally integrated the local and global features of the FVEP to make a decision.

There is a relatively small body of literature that is concerned with distinct characteristics in the medical field. These applications have two distinct characteristics: (1) There is a large amount of domain knowledge in the medical field. (2) For each of these tests, there were many categories of diseases detected. However, there exist some diseases that are few in number and are difficult to collect. In summary, there are two problems with the existing studies. (1) How to improve the accurate recognition rate of retinitis pigmentosa (RP) diseases. (2) How to realize the detection of out of distribution samples. To improve the performance of the RP recognition model and to detect out-of-distribution samples, it is necessary to adopt some of the advanced methods.

## Materials and methods

We first designed an experimental paradigm to solve the RP recognition problem. Then, we collected the FVEP dataset. Next, we applied some of the feature engineering methods and the MCAC model to RP recognition.

### Dataset

**Fig. 2:**
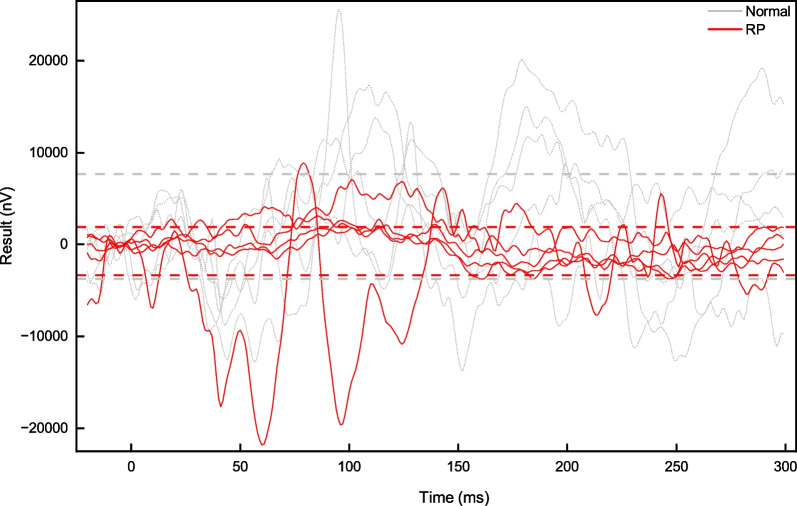
Visual FVEP Dataset

**Fig. 3:**
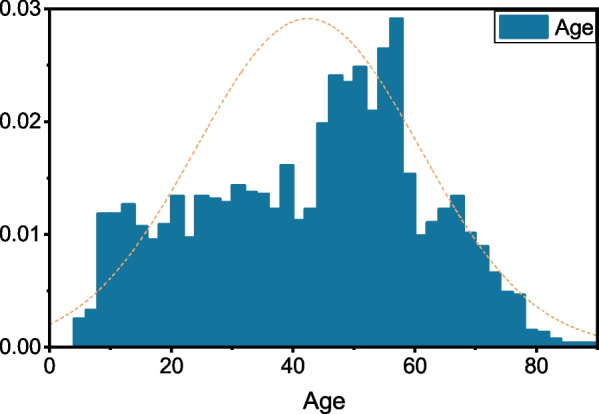
Age distribution of patients in the dataset

The FVEP dataset in this paper consists of four subdatasets; (1) a normal FVEP dataset, including 5164 FVEP data, collected from 1366 patients, each providing test results for both the left and right eyes twice; (2) an RP disease FVEP dataset, including 1112 FVEP data, collected from 278 patients, each providing test results for both the left and right eyes twice; (3) an abnormal FVEP dataset that includes a collection of apparently abnormal FVEP data and optic neuritis FVEP signals, totalling 800 items; and (4) an unlabelled FVEP dataset, including 4000 FVEP data, collected from 1000 patients. As shown in Fig. 2, the FVEP signals are different in normal people and RP patients. However, it is difficult to distinguish the RP disease FVEP signal from the normal human signal, as shown in Fig. 2 closest to the lower edge. In addition, patients were 4-88 years old, with an average age of 42 years (see Fig. 3).

All four datasets were collected from the Ophthalmology Department of the First Affiliated Hospital of Army Medical University (Southwest Hospital) from July 1, 2012 to March 1, 2020. The equipment used for FVEP collection was the Espion E2, which was loaded with the Espion E2 system.

### Feature engineering

With the development of deep neural networks, automatic feature extraction techniques are becoming increasingly mature. For example, recurrent neural networks and self-attentive networks can extract timedependent features in sequences, and convolutional neural networks can automatically extract pattern or spatial features in images. In some studies of time series classification problems, the manual features and depth features are utilized simultaneously to improve the classification accuracy [12, 13]. In this paper, the FVEP manual features extracted from the domain knowledge are combined with the deep features extracted from the neural networks to obtain more useful information for prescreening RP diseases. The manual features alone have difficulty capturing complex disease patterns, while the deep features are more comprehensive than the manual features [14].

**Fig. 4:**
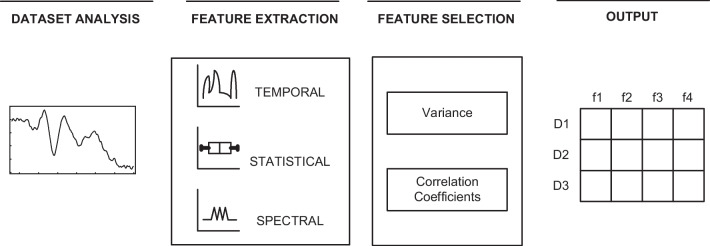
Feature engineering of FVEP signals

As shown in Fig. 4, feature engineering can be divided into three stages, including exploratory data analysis, feature extraction and feature selection. First, exploratory analysis of the dataset is performed using visualization tools to find information that can be used. Then, in this paper, time series feature extraction is performed by using domain knowledge with the help of the time series feature extraction librarytsfel tool (TSFEL). TSFEL includes over 60 different features extracted across temporal, statistical and spectral domains. Finally, feature selection is performed on the extracted features by using a feature selection algorithm.

As shown in Fig. 4, time series feature extraction can be divided into three categories, including the temporal, statistical and spectral domains. The temporal domain method mainly extracts time-related features from time-series data, including autocorrelations, mean differences, and entropy. The statistical domain method is mainly used to extract features by statistical methods, including the maximum, minimum, median, histogram, etc. The spectral domain method is mainly used to convert the FVEP signal to the spectral domain for feature extraction, including the fast Fourier transform [15], FFT mean coefficient, wavelet transform [16], etc.

In the feature selection stage, the variance filtering algorithm [17] and the Pearson correlation coefficient algorithm [18] are chosen. The variance filtering algorithm is based on the principle of calculating the variance corresponding to each feature value in the dataset and rejecting it if it is below the threshold. By default, all the zero-variance features will be rejected, and a variance of 0 means that the feature values of the sample have not changed. The Pearson correlation coefficient principle calculates the linear relationship between the features and labels, and rejects them if their values are close to zero.

**Fig. 5:**
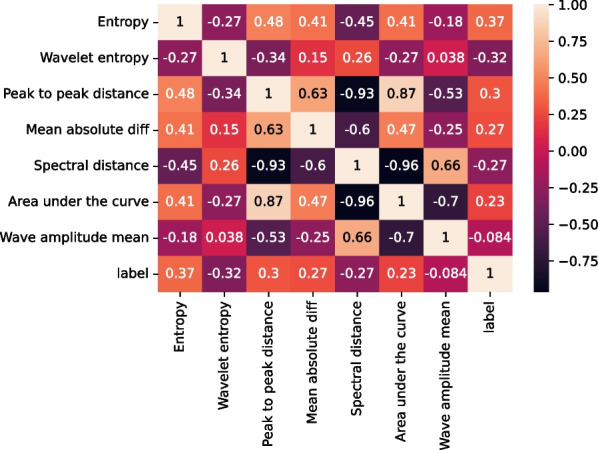
Correlation visualization

Figure 5 shows the correlation coefficient of the manual features. Some features were positively correlated with RP disease, and the others were negatively correlated with the RP disease. The FVEP of RP patients showed decreased amplitude and unchanged peak time in P2 wave. The reason is that generalized retinal dysfunction in RP will cause much smaller input for the following visual passway, while the conduction time of visual passway was usually unaffected. Therefore,the P2 wave in RP patients displayed decreased amplitude and unchanged peak time compared to the normal control.

### MCAC Model

**Fig. 6:**
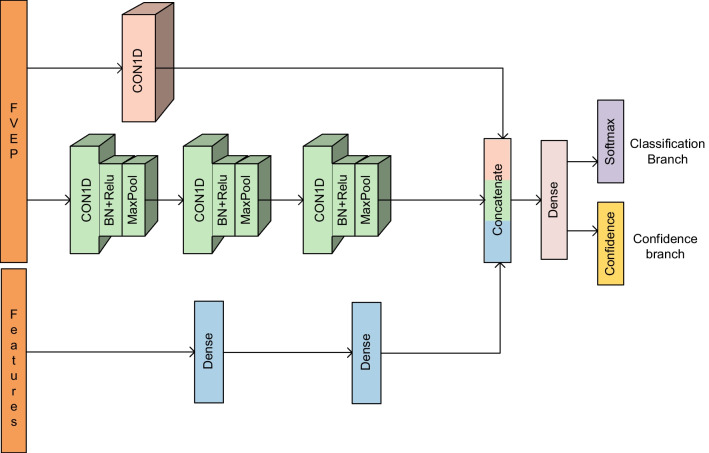
MCAC-Net architecture

Figure 6 presents our proposed multi-input neural network based on convolution and confidence branching (MCAC-Net). MCAC-Net has two inputs and two outputs, where the inputs include the FVEP signal and 7 manually extracted features, and the output includes the category and confidence level. For the manual feature input, the features are extracted through a fully connected layer of 128 neurons. For the FVEP signal input, the waveform features are extracted after two branches, i.e., the global feature extraction branch and the local feature extraction branch. For global feature extraction, a global one-dimensional convolution with a convolution kernel of the same size as the length of the FVEP signal is used. For local feature extraction, a one-dimensional convolution with a smaller convolution kernel size combined with maximum pooling is used. Finally, the outputs of the three branches are concatenated and passed through a layer of fully connected layers to extract features for classification and out-of-distribution detection. The network outputs categories with category confidence, and its category output is considered meaningful when the category confidence is greater than a certain threshold. The construction details of the network blocks are described as follows:

#### Global convolution

In the field of deep learning, RNNs were previously mainly used to capture temporal patterns or features. However, due to the inherent nature of RNNs, it is difficult to handle long time sequences and perform parallel computations, which ultimately affects the computational speed and model performance. The convolutional structures have demonstrated efficient parallel computations as well as the ability to capture features [19]. In this paper, we adopt a $\hbox{T}\times 1$ filter, called a global convolution, where T is the time length of the input FVEP signal and its value is 320. A global convolution extracts features from the integrated sequence at once, and will capture the nontime-invariance (time-invariance) features in the time series. Each global convolution filter processes the entire input and returns a vector of size with a RELU activation function. Integrating global convolutions will give the output. Each line of the output can be considered a representation of the entire time series.

#### Local convolution

The ability to have local patterns, considering the shorter time steps, is relevant for the predictions. Therefore, MCAC-Net utilizes a local convolution parallel to the global convolution to capture the local features. The focus of the two convolutions is inconsistent; the global convolution focuses on the features of the time series as a whole, while the local convolution focuses on the features of the sequence locally, and fusing the two features improves the classification performance. Unlike the global convolution, the length of the local convolution filter is small. To extract the most representative features, MCAC-Net utilizes a one dimensional maximum pooling layer. In this section, MCAC-Net utilizes a filter length of 3, including three convolutional layers and three maximum pooling layers.

#### Confidence branching

After global and local feature extraction, the manual features are fused and concatenated to make predictions. Unlike general classification tasks, MCAC-Net outputs category confidence c in addition to the category. When the category confidence is low,the category branch output is considered meaningless.

1 $$\begin{aligned} \text{p,c}={\mathcal{F}}\left( x,\theta \right) \quad {{\text{p}}_{i}},c\in \left[ 0,1 \right] ,\quad \sum \limits _{i=1}^{M}{\text{p}}_{i}=1 \end{aligned}$$

When we train MCAC-Net, some hints are provided to the network to adjust the softmax output using the confidence c.

2 $$\begin{aligned} {{\hat{p}}_{i}}=\text{c}*{{p}_{i}}+\left( 1-c \right) {{y}_{i}} \end{aligned}$$

Due to the imbalance between RP and the normal samples in the dataset, choosing focal loss as the loss function can reduce the data imbalance impact on the classification performance. After replacement using the new softmax output, the new loss function of MCAC-Net is:

3 $$\begin{aligned} {{L}_{t}}= & {} -\underset{i=1}{\overset{M}{\mathop \sum }}\,( y*{{\left( 1-{{{\hat{p}}}_{i}} \right) }^{\gamma }}*\log \left( {{{\hat{p}}}_{i}}\right) \nonumber \\{} & {} +( 1-y )*{{{\hat{p}}}_{i}}*\log ( 1-{{{\hat{p}}}_{i}})) \end{aligned}$$

4 $$\begin{aligned} {{L}_{c}}= & {} -\log \left( c \right) \ \end{aligned}$$

5 $$\begin{aligned} \text{L}= & {} {{\text{L}}_{t}}+\lambda {{\text{L}}_{c}}\ \end{aligned}$$

First, for the Focal Loss loss function ${{\text{L}}_{t}}$, ${{\hat{p}}_{i}}$ is used instead of ${{p}_{i}}$. Then, a confidence loss ${{L}_{c}}$ is added to prevent the neural network from always choosing $c = 0$ during training. finally, the ratio of the two losses is controlled using $\lambda$

#### Pretraining strategy

**Fig. 7:**
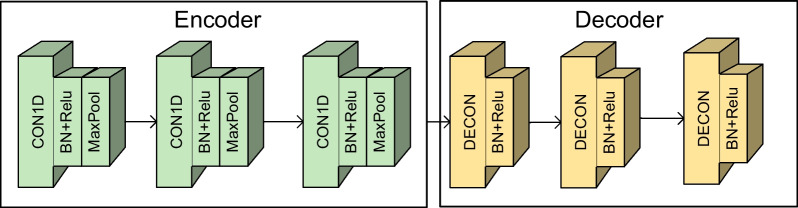
Pre-training Model

To utilize the unlabelled data in the dataset, this paper adopts a pretraining strategy for the local feature branches. As shown in Fig. 7, this paper builds a convolutional autoencoder to automatically extract the FVEP signal. In the training phase, this paper first uses the training set combined with a large amount of unlabelled data to train the local branches unsupervised. Then, the local branches are integrated into MCAC-Net, and the local branches are trained for the second time by setting a low learning rate. The whole training process is shown in Algorithm 1.

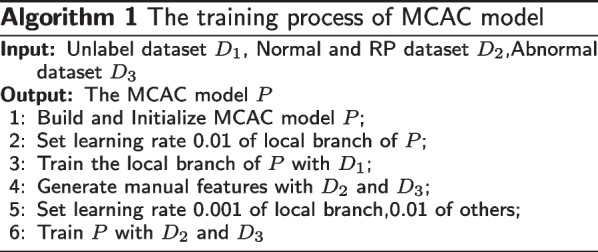



### Experimental setup

The experiment is divided into three parts. The first part compares the model classification performance, including comparing the performance of different neural network architectures when no manual features are added, versus observing the change in performance when the manual features are added. The second part compares the out-of-distribution detection performance of the different models. The third part provides a visual analysis. of the neural networks. In the first part of the experiment, for the MLP network, three fully connected layers are set, each with 128, 64 and 64 neurons in turn. for fully convolutional networks (FCN) [20], after the output of the full convolution layer, the fully connected layer is replaced with the global average pooling layer, which greatly reduces the number of parameters and avoids overfitting, and finally, the output is passed through the softmax layer. At the same time, the batch normalization and ReLU activation functions are used to accelerate convergence and reduce overfitting. The three convolutional layers are one-dimensional convolutions with filter sizes of 5, 3, 3 and the number of each layer is 64, 64, 64. For ResNet [21], three residual blocks are stacked. Each residual block consists of three convolutional layers with filter sizes of 5, 3, 3, and the number of each layer is 64, 64, 64. For the CNN LSTM network [22], the dropout layer is added in the LSTM branch to reduce overfitting, and the number of neurons in the LSTM is set to 64. In the CNN branch, after three convolutional layers, the global average pooling layer is connected, and then the outputs of the two branches are connected and output through the softmax layer. For the MCAC-Net network, we first temporarily remove its manual feature branch, which is CAC-Net. Unlike the Residual Network (ResNet), MCAC-Net uses maximum pooling for filtering to extract the more valuable features. The three convolutional layers in the local feature extraction branch of MCAC-Net have filter sizes of 5, 3 and 3, and the number of each layer is 64, 64 and 64 in that order. The convolutional layers in the global feature extraction branch have a filter size of 320 and a number of 64.

In the second part of the out-of-distribution detection experiments, we compare three types of models. First, the traditional anomaly detection models include LOF (local outlier factor). Breunig et al. [23], one class SVM [24], and a minimum covariance determinant (MCD) [25] model, are used based on the features output from the last fully connected layer of MCAC-Net. For these methods, the training set of the normal class is needed. In this paper, the training set of the two classes from the classification experiment is used as the out-of-distribution detection training set. Then, the information entropy of the MCAC-Net output is calculated, and if it is higher than a certain threshold, it is an out-of-distribution sample. This method does not require additional training. Finally, for the confidence-based algorithm, the training set from an anomalous FVEP dataset is needed in addition to the training sets of the two categories in the classification experiment

### Hyperparameters

The MCAC-Net2 model adopts pretraining technology and sets the local branch learning rate to 0.001. The remaining parameters are kept consistent with all the models, the learning rate is set to 0.01, the batch size is set to 128 and the number of iterations is 50. All the neural network models have focal loss as their loss function, which is expressed as eq. We select the model with the lowest training loss during training as the best model in the training set and report their test set evaluation results.

### Training and testing

For the normal FVEP dataset, the RP FVEP dataset was grouped according to the patients’ ID, data from 70% of the patients were randomly selected as the training set data, and data from 30% of the patients were used as the test set data. For the abnormal FVEP dataset, 30% of the data were randomly selected as the training set, and 70% of the data were used as the test set. The data of each patient includes the FVEP signal, age and disease type.

### Development environment

For the experiments in this paper, the computer configuration is composed of an AMD 2600 CPU, GTX1070 TI GPU and 16 GB of RAM. The data preprocessing, manual feature extraction, and MCSA-Net models are run on the Windows 10 64-bit operating system, and the deep learning framework used is Tensorflflow 2.0, executed in the Anaconda program.

## Evaluation criteria and results

### Evaluation criteria

The classification and out-of-distribution detection tasks are evaluated separately. To implement a comprehensive and objective evaluation of the model, we will evaluate the experiments using four different evaluation metrics. First, we introduce the basic few terms of the confusion matrix, true positives (TP) for the positive classes judged as positive classes, false-positives (FP) for the negative classes judged as positive classes, false negatives (FN) for the positive classes judged as negative classes, and true negatives (TN) for the negative classes judged as negative classes. The specific five metrics are shown below.

Accuracy (Acc): indicates the number of correctly predicted samples/total number of samples. Acc represents the overall classification performance of the model.

Precision: Precision indicates the proportion of the samples determined to be positive classes that are true positive classes. It measures how many of the positive classes predicted by the model are wrong.

Recall (Recall): Recall indicates the proportion of positive class samples that are judged to be positive and measures the model’s ability to check the positive class.

### Results for classification

In this section, the purpose of the experiment is to compare the classification performance of different models. In Table 1, we provide five metrics to fully evaluate the different models. The bolded cells are cells that outperform the state-of-the-art model. The performance in Table 1 indicates the superiority of MCAC-Net2 over the existing state-of-the-art models.

**Table 1:** The experiment results of classification

	Accuary	Precision	Recall	F1
BP	0.776	0.844	0.885	0.864
FCN	0.797	0.848	0.910	0.878
ResNet	0.801	0.861	0.897	0.879
CNN-LSTM	0.799	0.857	0.901	0.878
CAC-Net	0.810	0.864	0.906	0.885
MCAC-Net1	0.891	0.942	0.921	0.931
MCAC-Net2	**0**.**907**	**0**.**944**	**0**.**940**	**0**.**942**

Bold indicates optimal values

**Fig. 8:**
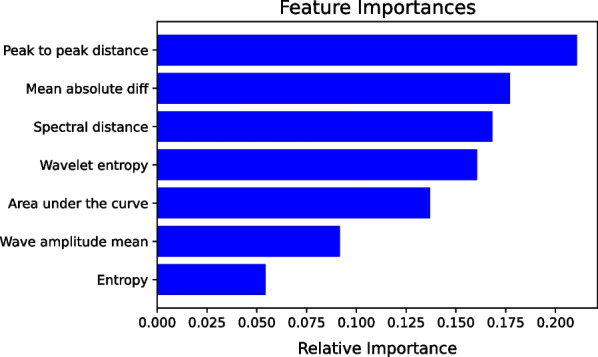
Manual feature importance visualization

The classification experiment is divided into three subexperiments. First, to compare network architectures, manual features were not added first. The results obtained from Table 1 show that the proposed CAC-Net has the best performance among the top five models that have no manual features. Then, to compare the change in model performance after adding manual features, we add manual features in the last two models. It can be seen from the data in Table 1 that MCAC-Net1 and MCAC-Net2 have significant performance improvements compared to CAC-Net. Finally, when we adopt prelearning technology, Table 1 shows a slight performance increase. Moreover, as shown in Fig. 8, the peak-to-peak distance feature has the highest importance value among the seven manual features,indicating that it is the most important for RP model identification.

### Results for out-of-distribution detection

In this section, the purpose of the experiment is to compare the out-of-distribution detection performance of different models. In Table 2, we provide five metrics to fully evaluate the different models. The bolded cells are cells that outperform the state-of-the-art model. The performance in Table 2 indicates the superiority of MCAC-Net over the existing state-of-the-art models.

**Table 2:** The experiment results of out-of-distribution detection

	Accuary	Precision	Recall	F1	Auc
Entropy	0.603	0.777	0.488	0.600	0.600
LOF	0.752	0.846	0.610	0.742	0.701
OCSVM	0.744	0.810	0.640	0.715	0.715
MCD	0.818	0.844	0.694	0.762	0.762
MCAC-Net	**0**.**933**	**0**.**868**	**0**.**905**	**0**.**891**	**0**.**835**

Bold indicates optimal values

It can be seen from the data in Table 2 that the traditional information entropy method based on the output of neural networks has the lowest accuracy for out-of-distribution detection. However, at the same time, it does not require additional training, and can be used as a baseline model for comparison. From Table 2, it can be seen that the performance of the model for out-of-distribution detection using traditional anomaly detection algorithms based on the features extracted by MCAC-Net is between the information entropy and the proposed model, and requires in-distribution samples for training.

## Conclusions

In this paper, an RP disease identification and out-of-distribution detection framework is highlighted to achieve a distinction between the normal classes as well as the RP classes and to perform out-of-distribution detection of the samples that do not belong to these two classes. First,we build a neural network model, MCAC-Net, that extracts the global and local features of the FVEP signal by relying on global and local convolutions, while incorporating a priori manual features, including several statistical features of the FVEP signal and age. Then, we compared different advanced time-series neural network architectures with the proposed architecture MCAC-Net. It was observed that MCAC-Net outperforms the other models, which were validated using the clinical dataset. Furthermore, other out-of-distribution detection algorithms were compared, and MCAC-Net outperformed the other models. The results suggest that our model contributes to the primary screening for RP disease.

## Discussion

Traditional deep learning models focus on classification accuracy, while in the clinical setting the complexity of diseases, with many types of diseases and uneven distribution, makes physicians urgently need a classification model with more generalization capability. For example, in the traditional binary classification model, samples that appear in the third category are recognized by the model as one of the two categories. DeVries T et al. proposed a method to add confidence branching with modified loss function to the existing neural network to achieve a fast scaling of the classification model and solve the out-of-distribution detection problem. Therefore, this paper proposes a confidence branching-based RP recognition and out-of-distribution detection model, which has the ability to recognize out-of-distribution samples and is experimentally proven to have excellent prediction accuracy.

In the visual electrophysiological examination, flash visual evoked potential (FVEP) varies greatly among individuals, resulting in different waveforms in different normal subjects. Moreover, most of the FVEP wave labelling was automatically performed by a machine, and sometimes was manually corrected by professional clinical technicians. These labels may have biases due to individual variations in the subjects, incomplete clinical examination data, different professional skills, personal habits and other factors. The labelling results can be very different and time consuming, which disrupts the clinical diagnosis process. The RP disease identification and out-of-distribution detection model developed in this paper has some limitations. There are many diseases related to vision that appear in patients in the clinic, not only RP diseases. Therefore, in future work, more types of optic nerve diseases should be classified. Additionally,physicians deciding whether there is an optic nerve disease should combine information such as the patient descriptions and imaging examinations, and to achieve more accurate classifications, classification models that handle multimodal data should be developed.

In the future, we will collect more RP disease data. The model in this paper identifies both normal and Rp categories, as well as out-of-distribution samples. By comparing the two sets of experiments, the F1-score of the proposed MCAC model for the classification experiment is significantly higher than the F1-score of the out-of-distribution identification experiment by 5.1%. In addition, the abnormal FVEP dataset contains only 800 samples, which has a large gap with the positive class samples. It can be seen that the two-category ratio of the out-of-distribution recognition experiment is more disparate compared to the two-category ratio of the classification experiment. In addition, there are many out-of-distribution sample categories, and this paper only includes several, and the proportion of each sample varies, further increasing the difficulty of out-of-distribution recognition, which leads to its low recognition performance. In the future, we will extend the model to be able to distinguish more classes of FVEP signals, and also generate more RP samples using generative adversarial networks.

## Data Availability

The use of data in this study is limited, and the data set can be obtained from the corresponding author (Chengliang Wang) according to reasonable requirements. Researchers or readers can send emails to corresponding author’s mailbox, and we will share the data without stint.

## Abbreviations

FVEP: Flash visual evoked potential
RP: Retinitis pigmentosa
MCAC-Net: A novel multi-input neural network based on convolution and confidence branching
CNN: Convolutional neural network
FCN: Fully convolutional networks
CAM: Class activation mapping
